# Successful Management of Disseminated Intravascular Coagulation in Metastatic Castrate-Resistant Prostate Cancer With Lutetium-177: A Case Report and Review of the Literature

**DOI:** 10.7759/cureus.75107

**Published:** 2024-12-04

**Authors:** Jie Zheng, Chidera Ibezue, Nghi Nguyen, Qi Cai, Qian Qin, Jue Wang

**Affiliations:** 1 Department of Internal Medicine, University of Texas Southwestern Medical Center, Dallas, USA; 2 Department of Internal Medicine, University of Texas Southwestern Medical School, Dallas, USA; 3 Department of Radiology, University of Texas Southwestern Medical Center, Dallas, USA; 4 Department of Pathology, University of Texas Southwestern Medical Center, Dallas, USA; 5 Department of Internal Medicine, Division of Hematology and Oncology, University of Texas Southwestern Medical Center, Dallas, USA

**Keywords:** androgen receptor (ar), circulating tumor dna (ctdna), disseminated intravascular coagulation, gene amplification, lutetium-117 vipivotide tetraxetan, prostate cancer

## Abstract

Disseminated intravascular coagulation (DIC) is a hematological disorder characterized by the abnormal activation of the coagulation system, which leads to widespread clotting and subsequent consumption coagulopathy. DIC is often associated with the progression of prostate cancer and can be a life-threatening condition. In this case report, we present a patient with recurrent DIC in the setting of advanced prostate cancer. His initial episode of DIC was effectively managed with a triple therapy regimen of leuprolide, docetaxel, and darolutamide. However, during a subsequent episode of DIC, which occurred with progression to widespread bone metastases, a single dose of Lu-177 vipivotide tetraxetan successfully resolved the condition. Circulating tumor DNA testing at the onset of the second episode of DIC revealed high levels of androgen receptor, CCND1, and PDGFRA gene amplification. This case not only underscores the critical importance of awareness, early diagnosis, and the prompt initiation of effective cancer therapy for managing life-threatening DIC associated with prostate cancer but also offers new molecular insights into the condition.

## Introduction

Prostate cancer is the most common cancer other than skin cancer in men worldwide and is the third leading cause of cancer-related deaths. In the United States, there are approximately 299,010 new cases and 35,250 deaths in 2024, and it continues to be the second leading cause of cancer-related death in American men [1].    

The mainstay of treatment of advanced prostate cancer is androgen deprivation therapy (ADT) plus novel androgen receptor pathway inhibitors with or without chemotherapy. However, with progression of disease, prostate cancer becomes hormone-resistant with more cancer-related complications [2]. One of the common hematological complications is disseminated intravascular coagulation (DIC) [3,4].   While exact prevalence rates are not well-defined, DIC is recognized as a significant complication in patients with metastatic castration-resistant prostate cancer (mCRPC), often associated with poor prognosis and requiring prompt and aggressive management.

DIC is a pathologic systemic condition secondary to aberrant activation of the coagulation system, characterized by coagulopathy, hypofibrinogenemia due to excessive fibrinolysis, and systemic bleeding [5,6]. Laboratory work is manifested with a significant decrease in platelet and fibrinogen as well as an increase in D-dimer and prothrombin time (PT)/partial thromboplastin time (PTT)/international normalized ratio (INR) by the International Society on Thrombosis and Hemostasis (ISTH) diagnostic criteria [6]. The incidence of DIC is 13-30% in metastatic prostate cancer [4,5,7] and it has been proposed to be an indicator of disease progression [4,8]. DIC secondary to advanced prostate cancer is a complex and life-threatening condition with poor prognosis. The management of DIC in prostate cancer involves addressing both the underlying malignancy and the coagulation abnormalities [7,8]. Case reports have demonstrated successful management of DIC using treatments such as degarelix [5,9], bicalutamide [10], and docetaxel [11,12].    

Lutetium-177 (Lu-177) vipivotide tetraxetan (Pluvicto®, formerly known as lutetium-177 PSMA-617) is a novel radioligand therapy designed to target prostate-specific membrane antigen (PSMA)-expressing prostate cancer cells. Administered intravenously, Lu-177 vipivotide tetraxetan binds to prostate cancer cells that express PSMA, a transmembrane protein found on the surface of these cells. Once bound, the beta particles emitted by the radioligand damage the cancer cells, which inhibits their replication and induces cell death [13]. While Lu-177 vipivotide tetraxetan is a promising treatment for mCRPC, its use in treating mCRPC associated with DIC has not been reported. In this case report, we described a case of recurrent DIC caused by prostate cancer progression that was successfully treated with a single cycle of Lu-177 vipivotide tetraxetan which led to prompt control of the patient's coagulopathy within two weeks of treatment. Our case highlights the interplay between androgen receptor (AR) amplification, prostate cancer progression, resistance, and coagulation, and the need for comprehensive management strategies that address both oncogenic signaling and coagulation abnormalities in advanced prostate cancer.

## Case presentation

**Figure 1:**
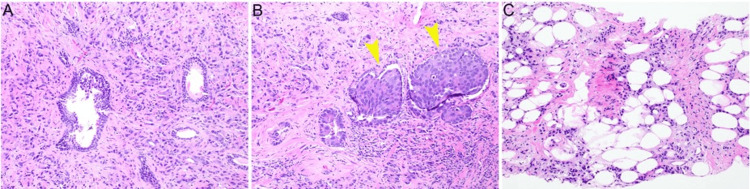
Representative morphology of prostate biopsy. A. Transrectal ultrasound-guided prostate biopsy specimen (hematoxylin and eosin 1000X) shows high-grade prostatic adenocarcinoma with Gleason score 5+4=9 (Grade Group 5); B. Intraductal carcinoma of the prostate (IDC-P) and adjacent invasive carcinoma (arrow); C. Tumor extended to extraprostatic adipose tissue (100 x magnification).

A 75-year-old male patient with a past medical history of hyperlipidemia, obstructive sleep apnea, and erectile dysfunction presented in November 2023 with rapidly rising prostate-specific antigen (PSA, from 41.17 ng/mL to 81.52 ng/mL within the span of three weeks). He underwent transrectal ultrasound-guided prostate biopsy in early January 2023, which showed prostate adenocarcinoma, 4+5 and 5+4=9, Grade Group 5 in 12/12 cores, extra prostatic extension, and perineural invasion (Figure 1).

Two weeks after prostate biopsy, he accidentally bit his tongue which caused an elongated course of tongue bleeding. He was evaluated by an Ear, Nose, and Throat (ENT) specialist. However, the bleeding was unable to be controlled by cauterization, and he was admitted to the hospital. Laboratory studies showed anemia, severe thrombocytopenia, elevated PT/PTT, decreased level of fibrinogen, and PSA of 105.22 ng/mL (Table 1).

**Table 1:** Disseminated intravascular coagulation (DIC) panel and prostate-specific antigen (PSA) before and one month after prostate cancer treatment with a triplet regimen (leuprolide, docetaxel, and darolutamide).

DIC Variables (Normal Range)	Before Triplet Treatment	One Month after Triplet Treatment
Platelets (150-450/μL)	73	209
Prothrombin Time (9.5-12.8 secs)	21.6	10.0
International Normalization Ratio (0.9-1.3)	1.9	1.0
Partial Prothrombin Time (23.4-34.3 secs)	48.6	N/A
D-dimer (<=0.59 mg/L)	N/A	N/A
Fibrinogen (214 - 459 mg/dL)	133	349
Prostate-specific antigen (ng/mL)	97.89	1.49

**Figure 2:**
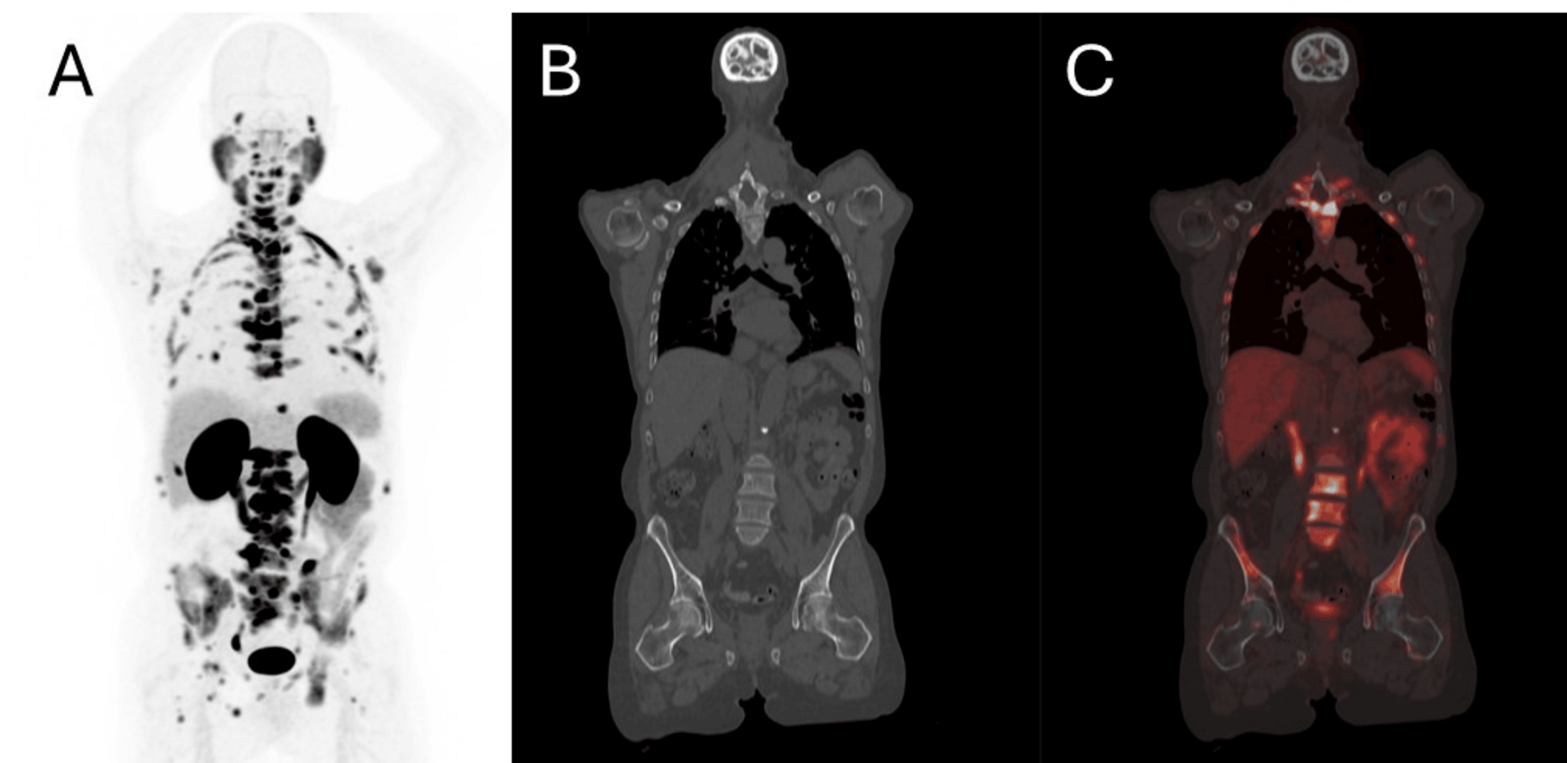
PET before Lu-177 vipivotide tetraxetan. Pre-therapy imaging. A. 3D PET image; B. Coronal CT image; C. Fused PET/CT image. Baseline diagnostic imaging with Ga-68 prostate-specific membrane antigen-11 positron-emission tomography/computed tomography (Ga-68 PSMA-11 PET/CT) revealed widespread bone metastases within the axial and appendicular skeleton (dark areas in A; bright thermal areas in C).

A Ga-68 PSMA-11 positron-emission tomography/computed tomography (PET/CT) scan showed diffuse disease within the prostate, extensive pelvic and retroperitoneal adenopathy, and widespread osseous metastasis. He received two units of platelet, three units of cryoprecipitate, and two units of fresh frozen plasma and was initiated on triplet therapy with leuprolide, docetaxel, and darolutamide. The PSA level decreased from 98 ng/mL to 1.49 ng/mL within one month of therapy initiation and reached an undetectable level after six months of therapy. The DIC panel also significantly improved with normalization of the fibrinogen level (Table 1). Despite this, PSA restarted to rise to 0.79 ng/mL on the eleventh month into treatment. The repeat PET/CT scan showed an overall response to treatment but more intense radioactive uptake over T10-T11 vertebral bodies and right manubrium, for which he received radiation therapy. Unfortunately, he developed progressive disease and PSA rose to 6.62 ng/mL at 14 months into treatment. Treatment was switched to abiraterone/prednisone without response and PSA rose to 15.5 ng/mL. Repeat Ga-68 PSMA-11 PET/CT showed worsening of his widespread osseous metastatic disease (Figure 2).

He was not eligible for an ongoing clinical trial due to compression fracture; thus after discussion, he elected to proceed with Lu-177 vipivotide tetraxetan due to rapid progression of disease and symptomatic cancer-associated bone pain.    

Three days prior to scheduled Lu-177 vipivotide tetraxetan therapy, laboratory studies showed anemia with hemoglobin 10.5 (normal 12.4-17.3 g/dL) and severe thrombocytopenia with a platelet of 55 (normal 150-450/μL). Further investigation showed PT 14.0 (normal 9.5-12.8 second), INR 1.4 (normal 0.9-1.3), D-dimer >35.2 (normal<=0.59 mg/L), LDH 1212 (normal 125-220 U/L), and fibrinogen 82 (normal 214-459 mg/dL), and repeat PSA has risen to 25.79 ng/mL (Table 2).

**Table 2:** Disseminated intravascular coagulation (DIC) panel and prostate-specific antigen (PSA) before and after one dose of lutetium-177 vipivotide tetraxetan.

DIC variables (normal range)	Before Lu-117	After one dose of Lu-117
Platelets (150-450/μL)	55	164
Prothrombin Time (9.5-12.8 secs)	14.0	10.7
International Normalization Ratio (0.9-1.3)	1.4	1.0
Partial Prothrombin Time (23.4-34.3 secs)	30.6	27.1
D-dimer (<=0.59 mg/L)	>35.20	10.22
Fibrinogen (214 - 459 mg/dL)	82	246
Prostate-specific antigen (ng/mL)	25.79	10.36

**Figure 3:**
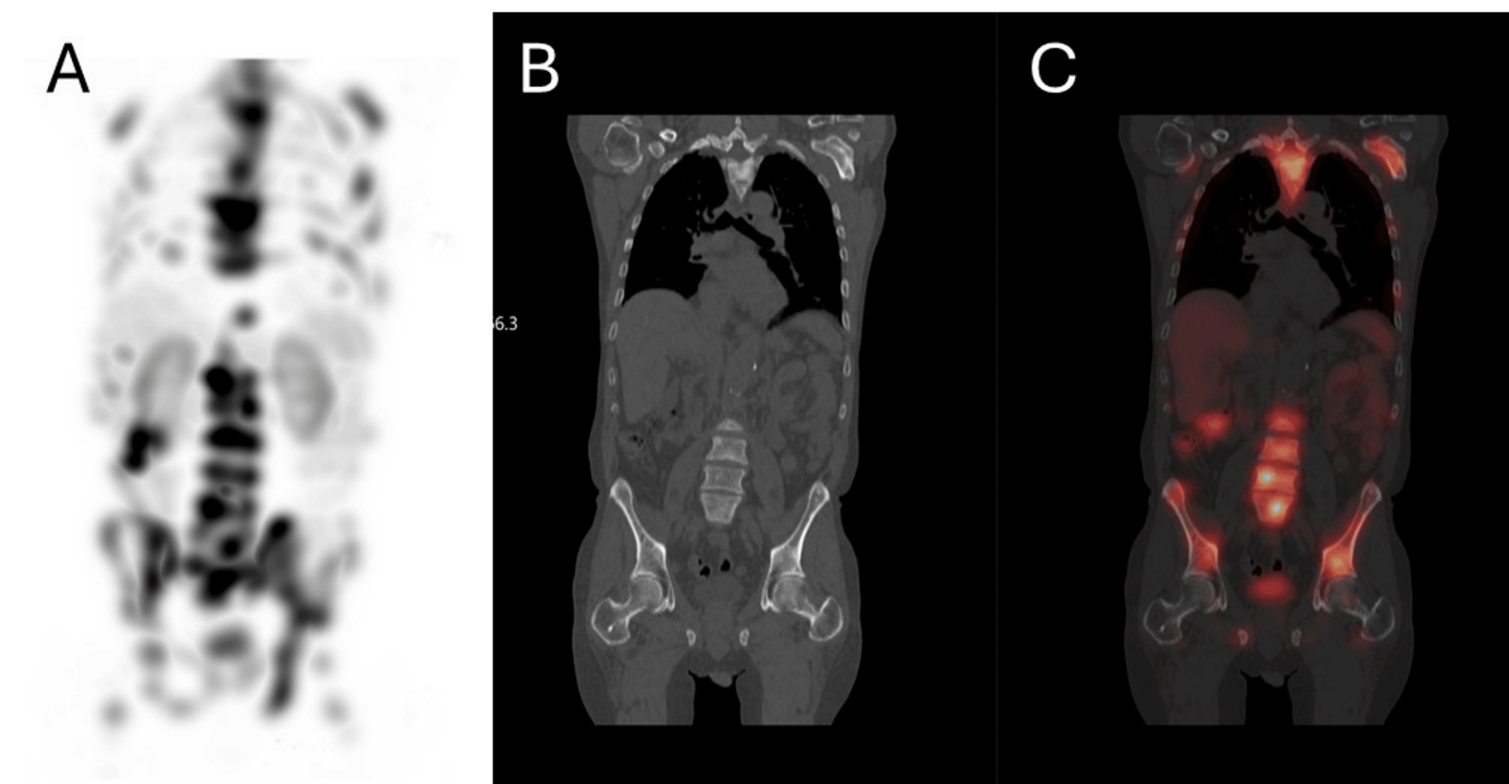
PET after one dose of Lu-177 vipivotide tetraxetan. Post-therapy imaging. A. 3D SPECT image; B. Coronal CT image; C. Fused SPECT/CT image. Single-photon emission tomography/computed tomography (SPECT/CT) imaging was performed two days following the IV administration of 206 mCi of Lu-177 vipivotide tetraxetan, demonstrating radioligand delivery to the osseous lesions, consistent with the radiotracer distribution previously seen on pre-therapy PET/CT imaging. The combination of Ga-68 PSMA-11 PET/CT imaging and Lu-177 vipivotide tetraxetan therapy represents a unique theragnostic approach pairing a diagnostic biomarker with a therapeutic radioligand that shares a molecular target of the cancer tissue.

Laboratory findings were consistent with prostate cancer-associated chronic DIC. Clinically, he did not show symptoms or signs of active bleeding or blood clotting. He was given one unit of cryoprecipitate and received 7.622 Gbq (206 mCi) of Lu-177 vipivotide tetraxetan on the same day, with post-therapy SPECT/CT imaging revealing radioligand distribution in osseous metastasis similar to that seen on diagnostic Ga-68 PSMA-11 PET/CT (Figure 3).

Two weeks after his first dose of Lu-177 vipivotide tetraxetan, he reported a resolution of bone pain and improved appetite. PSA decreased to 10.36 ng/mL from 25.79 ng/mL. Laboratory results also showed resolution of DIC (Table 2). The Lu-177 vipivotide tetraxetan is FDA-approved for up to six cycles, with 7.4 Gbq IV administration every six weeks.

**Figure 4:**
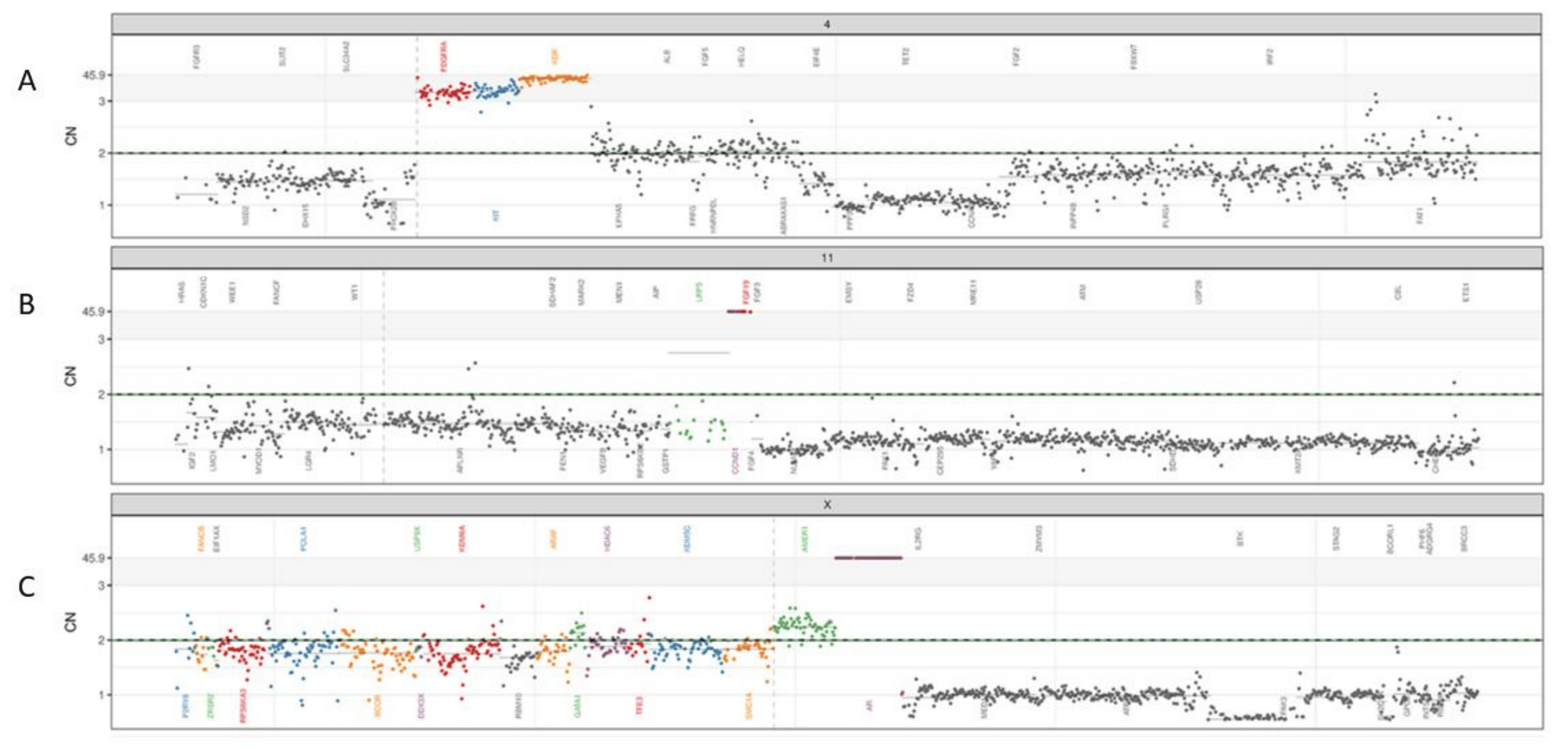
Circulating tumor DNA (ctDNA) testing reveals high levels of androgen receptor (AR), CCND1, and PDGFRA gene amplification. Plots show (A) PDGFRA (chromosome 4), (B) CCND1 (chromosome 11), and (C) AR (chromosome X) copy number compared to other detected genes.

Comprehensive genomic profiling of tumor tissue showed TMPRSS2-ERG chromosome rearrangement, PTEN copy number loss, tumor mutational burdens 2.1, and microsatellite instability (MSI) stable. Germline testing was negative. Further liquid biopsy for circulating tumor DNA (ctDNA) analysis at the time of DIC diagnosis showed high levels of AR amplification, Cyclin D1 (CCND1) and platelet-derived growth factor receptor alpha (PDGFRα) amplification (Figure 4).

## Discussion

DIC is a systemic condition characterized by widespread activation of clotting factors, which leads to the formation of fibrin within blood vessels and subsequent thrombotic occlusion. This process depletes coagulation factors and platelets, often resulting in life-threatening bleeding [7,14]. Patients with DIC typically exhibit three major features: coagulopathy, hypofibrinogenemia, and systemic bleeding [8]. The condition can be triggered by various factors, with sepsis being the most common cause. Additionally, malignancies are often associated with DIC, including advanced prostate cancer [7,14]. 

The diagnosis of DIC is based on clinical findings and laboratory tests showing impaired clotting function. Key indicators include prolonged activated PTT and PT/INR, low platelet count, reduced fibrinogen levels, and elevated levels of fibrin degradation products or D-dimer. Due to the absence of a specific test for DIC, the ISTH developed a scoring system in 2001 to aid in diagnosis. A score of 5 or higher on this system is suggestive of DIC [6]. 

Prostate cancer can be complicated by various hematological disorders, including DIC, thrombotic thrombocytopenia, primary fibrinolysis, thrombosis, thrombotic purpura, Trousseau’s syndrome, and acquired factor VIII inhibitor development. Among these, DIC is the most common coagulation complication in prostate cancer patients, with reported incidence ranging from 13% to 30%. However, only 0.4% to 1.65% of prostate cancer patients exhibit overt clinical signs of DIC, such as ecchymosis, petechiae, blood oozing from the mucosal surface or wound sites, or peri-operative bleeding [15]. 

The pathophysiology of DIC in prostate cancer remains not fully understood but is believed to be multifactorial. One key factor is tissue factor (TF), a procoagulant molecule expressed on the cancer cell surface membrane. TF forms a complex with factor VII, triggering a coagulation cascade that can lead to DIC [16]. Additionally, cancer cells express a molecule known as cancer procoagulant, a calcium-dependent cysteine protease that directly activates factor X, further driving the coagulation cascade. Prostate cancer cells uniquely secrete prostasomes, extracellular vesicles that express long chains of polyphosphates on their surface membranes. These polyphosphates activate the coagulation cascade predominantly through the intrinsic pathway, contributing to the development of DIC in advanced prostate cancer. This mechanism differs from DIC in other cancers, such as pancreatic or lung cancer, where DIC is often driven by overexpression of tissue factor, activating the extrinsic coagulation pathway. Highlighting this distinction underscores the specificity of prostate cancer-associated DIC and emphasizes the need for targeted research to develop tailored therapeutic approaches [17]. Finally, alterations of oncogenes and loss of tumor suppressor genes can influence angiogenesis, proliferation, and inflammation and are increasingly recognized as driving forces for hypercoagulation [18]. Our previous study explored the interplay of cancer progression and development of DIC using ctDNA assay [19]. The accumulation of specific genetic alterations, particularly the amplification of AR, oncogenes, and tyrosine kinase receptor genes, coincides with the onset of DIC in prostate cancer patients and implies a link between the tumor’s genetic evolution and the development of coagulopathy, highlighting the importance of early detection, monitoring, and targeted treatment strategies [19]. In this case, genomic profiling using ctDNA revealed a high level of AR amplification and several growth factor receptors at the time of onset of DIC, which was not present at the time of initial diagnosis, providing a potential mechanism of DIC in mCRPC. These insights align with the growing understanding of the complex interactions between prostate cancer progression, particularly in the context of AR and growth factor receptor amplification, and the promotion of coagulation abnormalities such as DIC [16,18,19]. AR alteration including amplification represents a significant genomic event during cancer progression to a castration-resistant state, which is common in advanced prostate cancer and contributes to treatment resistance and aggressive disease behavior [20]. AR amplification in prostate cancer cells can indeed lead to increased expression of androgen-responsive genes, which not only drive tumor growth but also contribute to the secretion of pro-coagulant extracellular vesicles (EVs). These EVs can carry a range of proteins, including TF and other pro-coagulant molecules, which may significantly enhance thrombin generation and activate the coagulation cascade [16]. This pro-thrombotic environment can lead to hypercoagulation states, increasing the risk of thromboembolic events and contributing to the pathogenesis of DIC in advanced prostate cancer. As we continue to explore these relationships, there is potential to improve the management and outcomes for prostate cancer patients at risk for DIC, moving closer to a precision medicine approach in oncology. Additionally, this knowledge reinforces the importance of monitoring coagulation parameters and potentially implementing prophylactic anticoagulation strategies in high-risk patients. 

DIC in prostate cancer is a critical indicator of disease progression and a poor prognostic factor, often heralding a terminal phase of the illness. If left untreated, DIC is a life-threatening condition with a high risk of hemorrhage and a very short survival time, and the urgency of early detection and treatment cannot be overstated [8,18]. Several case reports documented successful management of DIC in prostate cancer patients through various treatments including bicalutamide [3,10], degarelix [5,9], and docetaxel [11,12]. In our case, the first episode of DIC occurred following a prostate biopsy, manifested with tongue bleeding that could not be managed by supportive treatment or cauterization. The rapid initiation of prostate cancer triplet regimen with leuprolide, docetaxel, and darolutamide promptly controlled DIC, with a significant decrease in the PSA level. Unfortunately, the patient experienced a second episode of DIC fourteen months later due to disease progression. Prompt recognition of DIC and initiation of Lu-177 vipivotide tetraxetan lead to control of prostate cancer and by extension, management of the DIC. The choice of Lu-177 vipivotide tetraxetan was based on the patient’s PSMA-11 PET/CT scan showing PSMA avid disease and the VISION trial, an international, prospective, open-label, multicenter, randomized study evaluating Lu-177 vipivotide tetraxetan plus standard of care or standard of care alone in mCRPC patients. The results showed that the addition of Lu-177 vipivotide tetraxetan led to a statistically significant improvement in overall survival (median 15.3 months versus 11.3 months) and radiographic progression-free survival (median 8.7 months versus 3.4 months) compared to the control group [13]. The combination of Ga-68 PSMA-11 PET/CT imaging and subsequent Lu-177 vipivotide tetraxetan therapy represents a unique theragnostic approach pairing a diagnostic biomarker with a therapeutic radioligand that shares a molecular target of the cancer tissue. To the best of our knowledge, this is the first reported case of successful management of DIC with Lu-177 vipivotide tetraxetan in mCRPC. After just a single dose of Lu-177 vipivotide tetraxetan, the DIC panel results normalized, the PSA level decreased, and clinical symptoms improved. In our case, the normalization of DIC parameters was observed within 14 days following the administration of Lu-177 vipivotide tetraxetan, accompanied by clinical improvement. Reports from other studies suggest that the time to resolution of DIC can vary significantly depending on the treatment modality and the underlying malignancy. For instance, ADT and chemotherapy have shown responses within 1-2 weeks in some cases, although this can depend on the extent of disease burden and patient condition [3,5,9-12,19]. It is also worth noting that supportive measures, such as transfusions, may provide temporary stabilization but do not directly address the underlying cause, potentially prolonging the course of DIC.

While this case report offers valuable clinical insights into the management of DIC in prostate cancer, its limitations must be acknowledged. The focus on a single patient, lack of comparative data, and absence of long-term follow-up restrict the generalizability of the findings. Further research is needed to better understand and manage this challenging complication.

## Conclusions

In conclusion, DIC is a life-threatening condition in prostate cancer patients that requires prompt intervention. Active cancer therapy has the potential to reverse DIC in refractory prostate cancer by targeting the underlying malignancy, offering hope even in the terminal stages of the disease. As the disease progresses from castration-sensitive to castration-resistant status, managing recurrent DIC becomes increasingly challenging due to the cancer’s reduced responsiveness to subsequent lines of therapy. Lu-177 vipivotide tetraxetan demonstrated a notable response in this context, suggesting that it should be considered in similar cases where therapeutic options are limited. Further clinical research is needed to validate these findings and inform individualized strategies for managing prostate cancer-associated DIC. Our case also underscores the critical need to understand the interplay between molecular events such as AR and other tyrosine kinase growth factor receptor amplification, prostate cancer progression, treatment resistance, and DIC. 
